# Do tradeoffs structure antibiotic inhibition, resistance, and resource use among soil-borne *Streptomyces*?

**DOI:** 10.1186/s12862-015-0470-6

**Published:** 2015-09-15

**Authors:** Daniel C. Schlatter, Linda L. Kinkel

**Affiliations:** Department of Plant Pathology, University of Minnesota, 1991 Upper Buford Circle, Saint Paul, MN 55108 USA

## Abstract

**Background:**

Tradeoffs among competing traits are believed to be crucial to the maintenance of diversity in complex communities. The production of antibiotics to inhibit competitors and resistance to antibiotic inhibition are two traits hypothesized to be critical to microbial fitness in natural habitats, yet data on costs or tradeoffs associated with these traits are limited. In this work we characterized tradeoffs between antibiotic inhibition or resistance capacities and growth efficiencies or niche widths for a broad collection of *Streptomyces* from soil.

**Results:**

*Streptomyces* isolates tended to have either very little or very high inhibitory capacity. In contrast, *Streptomyces* isolates were most commonly resistant to antibiotic inhibition by an intermediate number of other isolates. *Streptomyces* with either very high antibiotic inhibitory or resistance capacities had less efficient growth and utilized a smaller number of resources for growth (smaller niche width) than those with low inhibition or resistance capacities, suggesting tradeoffs between antibiotic inhibitory or resistance and resource use phenotypes.

**Conclusions:**

This work suggests that life-history tradeoffs may be crucial to the maintenance of the vast diversity of antibiotic inhibitory and resistance phenotypes found among *Streptomyces* in natural communities.

**Electronic supplementary material:**

The online version of this article (doi:10.1186/s12862-015-0470-6) contains supplementary material, which is available to authorized users.

## Background

Soil bacteria produce an astounding array of antimicrobial compounds [1] Antibiotic production is believed to provide a fitness benefit to the producer by inhibiting the growth of competing microbes [2–5]. In response, competitors may overcome inhibition by acquiring resistance to antibiotics through mutations or horizontal gene transfer [6, 7]. Antibiotic inhibition and resistance phenotypes are extremely diverse in natural habitats and highly variable among soil bacteria [4, 8]. However, how this diversity is maintained in soil microbial communities is not well understood. In particular, what limits the accumulation of antibiotic production and resistance genes among bacterial populations in soil?

Tradeoffs preclude an organism’s ability to optimize multiple traits [9, 10]. It is commonly assumed that because antibiotic production and resistance generally require energy expenditure they are accompanied by tradeoffs with growth or other fitness components. As a result of tradeoffs, antibiotic-producing microbes are expected to be out-competed by non-producing counterparts in the absence of susceptible competitors [11–13]. Similarly, microbes that carry resistance to antibiotics are expected to be out-competed by susceptible counterparts in the absence of antibiotic-producing competitors [11, 13, 14]. Although tradeoffs between growth and antibiotic resistance have been studied in clinical settings [14], the lack of analogous data for antibiotic production or resistance among naturally-occurring microbial populations limits our understanding of the dynamics of antibiotic inhibition and resistance within soil communities.

Tradeoffs associated with antibiotic production and resistance should be reflected in growth reductions for antibiotic-producing vs. non-producing bacteria in the absence of competition (ie. physiological tradeoffs; [12]) or, more generally, in negative relationships among distinct phenotypic traits (ie. life history tradeoffs; [9]). Since bacterial strains often produce and resist many antibiotics in tandem [15–17], tradeoffs that are important for the fitness of individuals are likely to encompass the total or cumulative profile of antibiotic production and resistance. For example, production of multiple antibiotic compounds is likely to require a greater total allocation of resources compared to production of a single antibiotic, whereas the costs of multiple resistances may depend on the specific resistance mechanisms (eg. multi-drug efflux pumps versus the accumulation of resistance mutations). At the same time, it has been hypothesized that the accumulation of antibiotic production and resistance among bacteria is driven by a coevolutionary arms race [13, 15]. Arms race dynamics are hypothesized to result in extreme traits in populations [18]. For example, among microbial pathogen populations, arms race coevolution is considered to be responsible for the accumulation of virulence and resistance [19–21], and may also generate a preponderance of bacteria with highly antagonistic or resistant phenotypes [13]. However, there are few data on the distribution of cumulative antibiotic inhibition and resistance phenotypes among soil bacteria, and evidence for significant fitness tradeoffs associated with antibiotic inhibitory or resistance phenotypes is limited.

*Streptomyces* are filamentous, Gram-positive bacteria that are prolific producers of antibiotics and a significant reservoir of antibiotic resistance in soils [16, 17]. Antibiotic production by *Streptomyces* is often dependent on resource availability [22–24]. Moreover, *Streptomyces* vary extensively in the resources on which they can grow and in their relative growth efficiencies on different resources [25], suggesting the potential for diverse competitive strategies. Fitness tradeoffs associated with antibiotic inhibition or resistance may limit the evolution of *Streptomyces* that are both highly antagonistic and growth efficient on a wide array of resources. However, specific data on the relationships among *Streptomyces* resource use, antibiotic inhibition, and antibiotic resistance from natural settings are needed to understand the potential for fitness tradeoffs to constrain the accumulation of antibiotic inhibition or resistance capacities in soil microbes.

In this work we hypothesize that there are life-history tradeoffs associated with the antibiotic inhibitory and resistance capacities of *Streptomyces* within natural soil populations. We examine patterns of antibiotic inhibition and resistance phenotypes among *n* = 263 *Streptomyces* isolates from natural soil settings in relation to their resource use on 95 sole carbon sources. Specifically, we 1) characterize antibiotic inhibition and resistance among *Streptomyces* and quantify inhibition and resistance phenotypes among isolates; 2) determine relationships between antibiotic inhibition, resistance, and resource use among *Streptomyces* isolates; and 3) explore evidence for tradeoffs among *Streptomyces* exhibiting distinct inhibition and resistance strategies. These data suggest that tradeoffs may be critical in structuring antibiotic production and resistance strategies among *Streptomyces* in soil.

## Methods

### Soil sampling, processing, and isolation

Soil samples were collected at the University of Minnesota Cedar Creek Ecosystem Science Reserve (www.cedarcreek.umn.edu), a NSF Long-Term Ecological Research site as described previously [4]. Three soil cores were taken from random locations within 1 m^2^ sections of two plots in experiment E001 (plots 08-A and 10-D) for a total of six soil cores. Soil cores were transported to the lab on ice and processed immediately. Soils were dried overnight under a double-layer of sterile cheesecloth, serially diluted in phosphate buffer (0.5 M K_2_HPO_4_, 0.4 M KH_2_PO_4_, pH = 7.0), and plated on oatmeal agar as described in Davelos et al. [4]. Plates were incubated at 28 C for 7 days and *Streptomyces* densities were estimated based on characteristic colony morphology. *Streptomyces* colonies were randomly picked with a sterile toothpick, purified, and stored in 20 % glycerol at -80 C for further study. Detailed information on soil collection, processing, and isolation is published elsewhere [4].

### Characterization of *Streptomyces* antibiotic inhibition and resistance

*Streptomyces* antibiotic inhibition and resistance profiles were determined against ten standard reference isolates described in Davelos et al. [26] Standard strains encompass genotypically and phenotypically diverse isolates collected from multiple locations in Minnesota. Moreover, these test stains differ in their antibiotic inhibition and resistance profiles so that they can differentiate up to 1024 different inhibition and resistance phenotypes in pair-wise assays [26]. Because *Streptomyces* inhibitory phenotypes may be adapted to inhibit local resource competitors at small spatial scales (within 1 m^2^; [5]) and isolates were collected from multiple different locations, standard test isolates provide an assessment of *Streptomyces* antibiotic inhibitory and resistance phenotypes that is independent from the location of origin of the tested isolates. *Streptomyces* isolates (*n* = 263) were characterized for antibiotic inhibition profiles by evaluating their ability to inhibit each standard isolate using an agar-overlay method [4]. Briefly, 10 ul of spore suspensions (~10^8^ spores/ml) were dotted onto 15 ml starch-casein agar (SCA). After incubation for 3 days at 28 C, isolates were killed by inverting each plate over a watch glass containing 4 ml chloroform. Plates were then left open in a laminar flow hood for 30 min to allow residual chloroform to evaporate, then overlaid with 15 ml of fresh 1 % water agar. After the agar solidified 100 ul of each test standard spore stock (~10^8^ spores/ml) was spread on the plate. Plates were incubated for 3 days at 28 C and the size of inhibition zones around dotted isolates were measured. Each *Streptomyces*-standard interaction was replicated three times and only inhibition zones greater than 1 mm were considered to be inhibitory. The same approach was used to characterize the collection of *Streptomyces* isolates for resistance to antibiotic inhibition by each standard isolate. Spatial variation in the inhibition and resistance characteristics of a subset of the isolates used in this work have been published previously [4], though their relationships with resource use have not been explored previously.

### Resource use

Resource use was evaluated on Biolog SF-P2 microplates (Biolog, Inc. Hayward, CA) using standard procedures as described in Schlatter et al. [25]. Measurement of growth in Biolog SF-P2 plates rely on the turbidity of each well (absorbance) as a measure of microbial growth, rather than a redox dye. Thus, growth measurements in Biolog SF-P2 plates offer a rapid and standardized means to quantify the cumulative reproductive output of *Streptomyces* isolates on a diverse array of sole carbon sources. Fresh spore suspensions of each *Streptomyces* isolate were quantified to an absorbance of 0.22 at 590 nm, diluted according to the manufacturer’s instructions, and inoculated into Biolog SF-P2 plates. Plates were incubated at 28 C for 3 days and the absorbance of each well was measured (590 nm). The absorbance (au) from the water control well was subtracted from all 95 substrate-containing wells. After correction, all wells with an absorbance <0.005 were adjusted to 0 prior to analyses. We defined niche width as the number of substrates on which an isolate could grow (positive absorbance after adjustments). Growth efficiency was defined as the mean growth on used substrates (mean of absorbance values >0). Variation in resource use of these isolates has been explored [25], yet their relationships with inhibition or resistance traits have not been previously evaluated.

### Statistical analyses

All statistical analyses were performed in the R statistical package (Version 3.0.2, [27]) or GraphPad Prism (Version 6; GraphPad Software, La Jolla California, USA). Spearman correlations and linear regression were used to test for relationships between *Streptomyces* inhibitory, resistance, and resource use phenotypes. Permutation tests (*n* = 10,000 permutations) were used for Spearman correlations to estimate the significance of relationships using the ‘coin’ package in R [28]. Student’s t-tests with Welch’s corrections were used to examine differences in resource use phenotypes between *Streptomyces* with antagonistic or resistant phenotypes and their non-antagonistic or resistant counterparts for each individual standard tested. Because antibiotic inhibitory phenotypes among *Streptomyces* fell into two distinct categories, those that did not inhibit any standards (non-inhibitors, *n* = 109) and those that inhibited all 10 standards (super-killers, *n* = 38), Student’s t-tests were used to compare differences in resource use of *Streptomyces* among these categories. Similarly, although antibiotic resistance was normally distributed, resource use among *Streptomyces* with low (resisting 3–5 standards, *n* = 58) and high (resisting 8–10 standards; *n* = 62) resistance capacities was also compared using Student’s t-tests. Further, the ability of *Streptomyces* belonging to high- versus low-inhibition or resistance categories to utilize each of the *n* = 95 individual carbon sources was compared using Pearson’s Chi-squared tests, where p-values <0.05 after adjustment for multiple comparisons using an FDR correction were considered statistically significant.

## Results

### *Streptomyces* inhibition and resistance

**Fig. 1:**
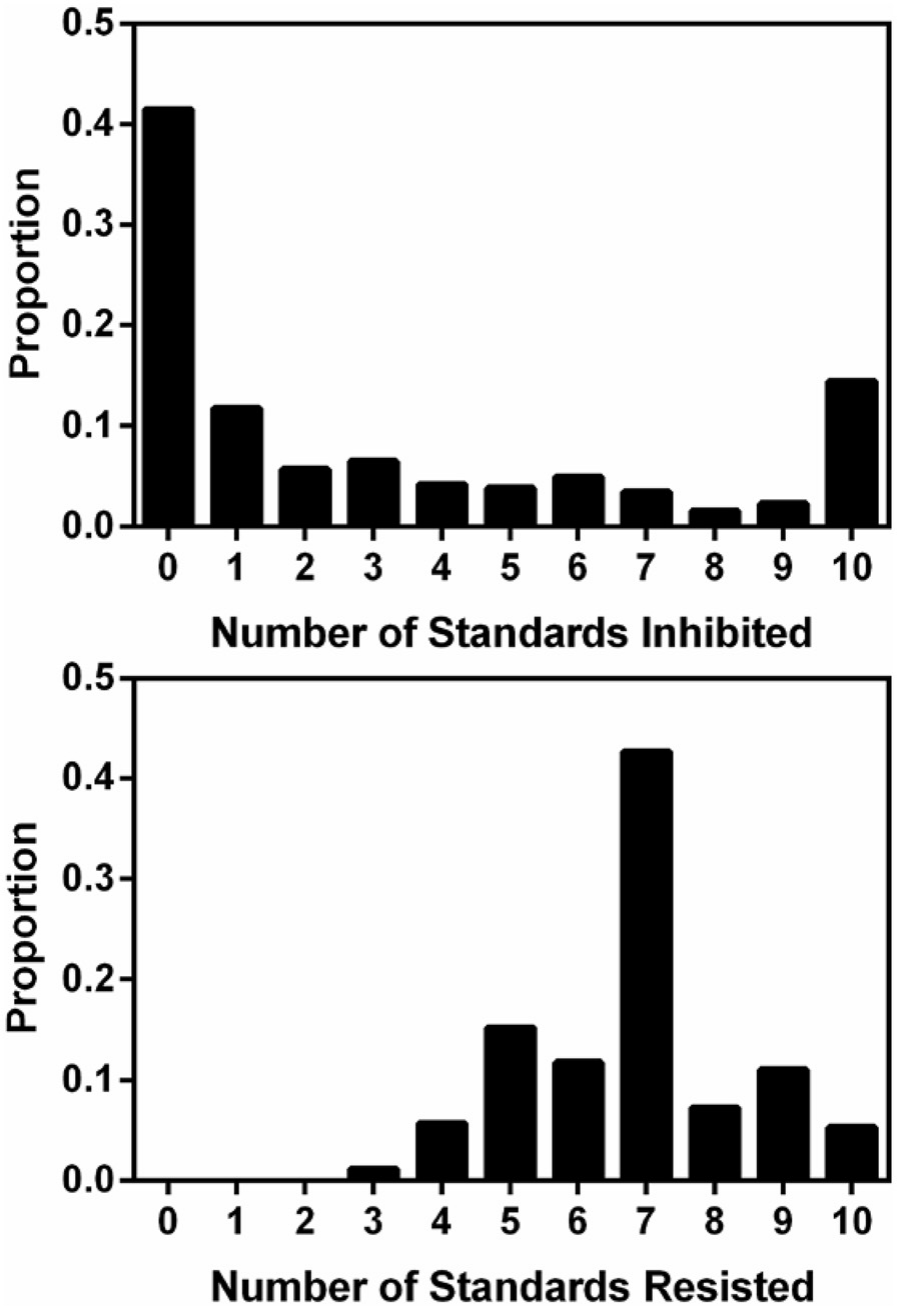
Frequency distribution of cumulative antibiotic inhibition (*upper panel*) and resistance (*lower panel*) phenotypes of *Streptomyces* against standard test isolates

*Streptomyces* isolates varied in their abilities to inhibit and resist standard strains, consistent with previous work [4]. Patterns of inhibition were diverse: considering discrete inhibitory phenotypes (+/−) there were 57 unique inhibition profiles among the 263 isolates. Overall, individual *Streptomyces* inhibited an average of 3.1 standards, though this average increased to 5.3 standards when considering only *Streptomyces* that could inhibit at least one standard. However, most isolates had either very little or very high inhibitory capacity, as reflected in the distinct bimodal distribution of isolate frequencies (Fig. 1a). The majority of *Streptomyces* either lacked any inhibitory capacity (41 % of isolates did not inhibit any standard; Fig. 1a) or had extremely broad inhibitory phenotypes (14 % of isolates inhibited all 10 standards; Fig. 1a).

Resistance phenotypes among *Streptomyces* were less diverse than inhibition phenotypes. Among 263 isolates there were 29 unique resistance profiles. *Streptomyces* isolates resisted 6.8 standards on average (Fig. 1b). Resistance to inhibition by multiple standards was common among soil-borne *Streptomyces*. Every standard inhibited at least two *Streptomyces* isolates, verifying that each standard had some inhibitory capacity. Although every *Streptomyces* isolate resisted inhibition by at least three standards, only a small proportion (5 %) of isolates was resistant to all ten standards. In contrast to the bimodal distribution of inhibitory phenotypes, frequencies of resistance were approximately normally distributed among isolates and fell around a distinct mode (median = 7, mode = 7).

*Streptomyces* capacities to inhibit and resist standards were weakly correlated. There was a marginally significant positive correlation between the number of standards inhibited and the number of standards resisted among individual *Streptomyces* (Spearman’s *ρ* = 0.15, *p* = 0.012, *z* = 2.49), suggesting that *Streptomyces* with broad antibiotic inhibition capacities are slightly more likely to have broad resistance capacities.

### Inhibition-resource use tradeoffs

*Streptomyces* with the ability to inhibit specific standards versus those with no apparent inhibitory capacity against that standard sometimes differed in growth efficiency or niche width (Table 1). Inhibitors of standards LK4-2, LK6-14, LK10-3, and DL87 had significantly smaller growth efficiencies than isolates unable to inhibit each of these standards. Similarly, inhibitors of standards LK4-2, LK4-21, LK6-14, LK10-3, and DL87 had significantly smaller niche widths than non-inhibitors. Overall, *Streptomyces* able to inhibit specific standards consistently had smaller niche widths than non-inhibitors against those standards (ten of ten standards), though differences were only significant for four of the ten standards. These results suggest that having inhibitory capacity towards a standard sometimes comes at a cost with respect to growth efficiency or niche width, though costs may vary among antibiotics.

**Table 1:** Differences in growth efficiency and niche widths among *Streptomyces* inhibitory and non-inhibitory towards individual standards

Standard	Growth Efficiency (± SD)					Niche Width (± SD)				
	Inhibitor	Non-Inhibitor	df	t-val	p-val	Inhibitor	Non-Inhibitor	df	t-val	p-val
LK2-12	0.067 ± 0.015	0.066 ± 0.016	158.1	0.52	0.605	70.5 ± 16.9	74.0 ± 16.2	140.9	−1.55	0.123
LK4-2	0.062 ± 0.012	0.068 ± 0.016	117.7	−3.17	0.002	67.1 ± 18.2	74.7 ± 15.5	82.5	−2.89	0.005
LK4-16	0.068 ± 0.017	0.066 ± 0.015	84.4	0.58	0.562	69.9 ± 18.0	74.1 ± 15.9	84.8	−1.63	0.108
LK4-20	0.066 ± 0.016	0.067 ± 0.015	132.6	−0.34	0.737	71.9 ± 16.3	74.1 ± 16.1	133.4	−0.99	0.325
LK4-21	0.065 ± 0.015	0.067 ± 0.015	114.1	−0.69	0.490	70.0 ± 17.4	74.3 ± 16.1	106.6	−1.77	0.080
LK4-24	0.066 ± 0.015	0.067 ± 0.015	149.4	−0.51	0.614	71.0 ± 16.9	74.0 ± 16.2	142.7	−1.31	0.191
LK6-14	0.063 ± 0.012	0.068 ± 0.016	158.2	−2.45	0.016	68.7 ± 16.8	74.7 ± 16.1	116.3	−2.59	0.011
LK10-3	0.063 ± 0.012	0.068 ± 0.016	144.3	−2.40	0.018	68.6 ± 16.5	74.5 ± 16.2	108.8	−2.50	0.014
DL87	0.063 ± 0.014	0.068 ± 0.016	209.4	−2.65	0.009	67.4 ± 17.3	76.2 ± 15.0	161.1	−4.09	<0.001
DL93	0.067 ± 0.016	0.066 ± 0.015	87.7	0.25	0.806	70.1 ± 18.0	73.8 ± 16.0	83.1	−1.38	0.170

Growth efficiency (±standard deviation) and niche width (±standard deviation) of inhibitory and non-inhibitory *Streptomyces* against individual standard isolates. Degrees of freedom, t-values, and p-values of t-tests between inhibitory and non-inhibitory *Streptomyces* are presented

**Fig. 2:**
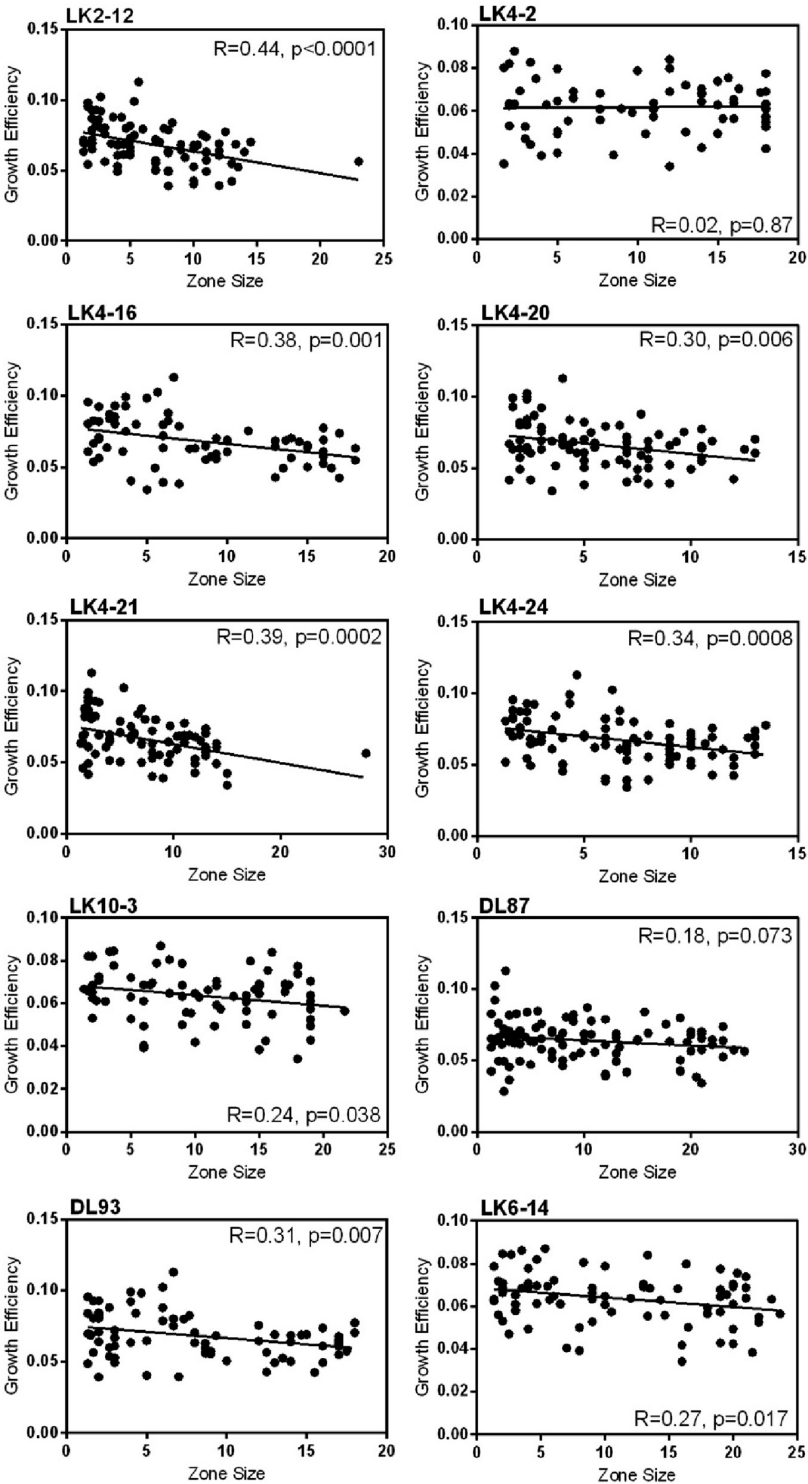
Linear regression of growth efficiency and inhibition zone sizes among inhibitors of each of the 10 standard isolates

Among *Streptomyces* that exhibited inhibitory capacities against particular standards, there were significant negative relationships between inhibition intensity (inhibition zone size) and growth efficiency for eight of ten standards (Fig. 2). Among isolates that could inhibit a given standard, inhibition zone size explained from 6 to 19 % of the variation in growth efficiency. This suggests that *Streptomyces* with stronger inhibitory capacities are less growth efficient, consistent with the idea that producing greater quantities of antibiotic imposes a cost in growth efficiency. Further, variation in the slopes of relationships between inhibition intensity and growth efficiency (Additional file 1) suggests that the costs of increased inhibition, or antibiotic production, vary among different standards. Relationships between inhibition intensity and niche width were also consistently negative, though statistically significant for only 5 of the 10 standards (Additional file 1).

**Fig. 3:**
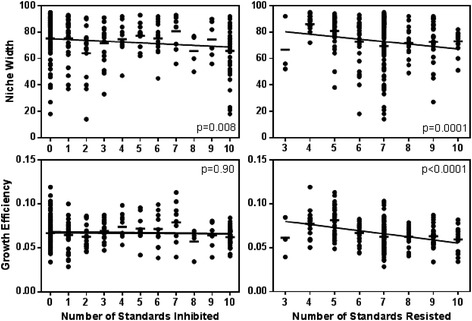
Relationships between cumulative inhibition (*left panels*) and resistance (*right panels*) phenotypes with niche width (*top panels*) and growth efficiency (*bottom panels*) among *Streptomyces* isolates. Spearman’s correlation coefficients and p-values are presented for relationships in each panel. Bars represent mean growth efficiency or niche width in each category

**Fig. 4:**
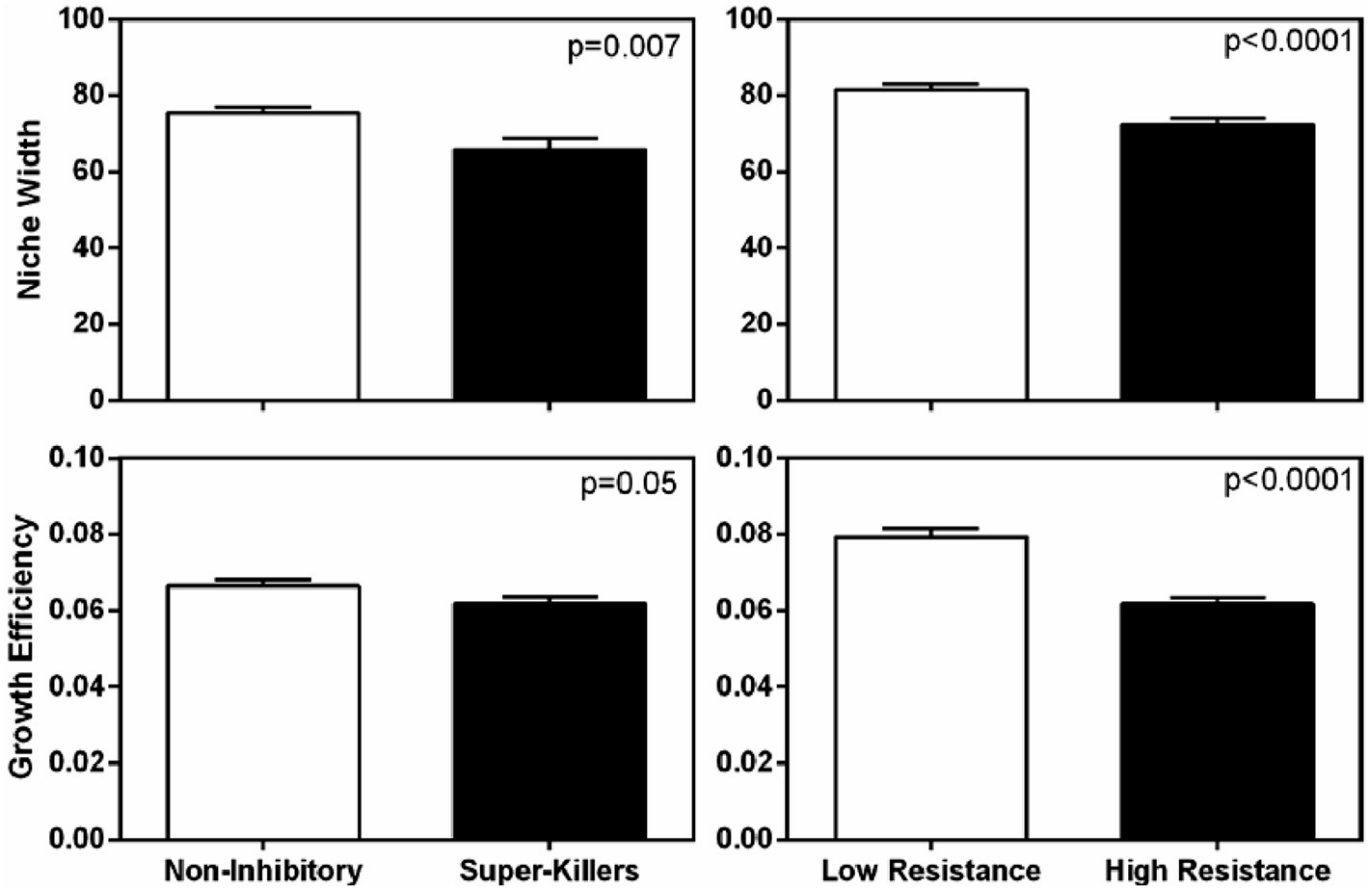
Niche width (±SEM; *top panels*) and growth efficiency (±SEM; *bottom panels*) among *Streptomyces* exhibiting distinct inhibition strategies (non-inhibitory vs super-killers; *left panels*) and resistance strategies (low resistance vs high resistance; *right panels*). P-values represent Student’s t-tests between groups in each panel

Considering cumulative inhibitory capacity, or the total number of standards that a *Streptomyces* isolate could inhibit rather than the inhibition zone size against a given standard, the number of standards that an isolate inhibited was weakly but significantly negatively correlated with niche width (Fig. 3; Spearman’s *ρ* = −0.16, *p* = 0.008, *z* = −2.64), but not growth efficiency (Fig. 3; Spearman’s *ρ* = 0.008, *p* = 0.90, *z* = 0.13). Thus, isolates that inhibited a larger number of standards had smaller niche widths than those that inhibited fewer standards. However, correlations between total number of standards inhibited and niche width were weak, perhaps due to the variation in the costs of producing diverse antibiotics and inhibiting different standards. Notably, non-inhibitory isolates (inhibited none of the standards) and highly inhibitory ‘super-killers’ (inhibited all 10 standards) differed significantly in resource use. Super-killer *Streptomyces* had significantly smaller niche widths and grew less efficiently than non-inhibitors (Fig. 4). Non-inhibitory *Streptomyces* had on average 12.7 % larger niche widths (*t*-test, *t* = 2.77, *p* = 0.007) and 6.8 % more efficient growth (*t*-test, *t* = 2.01, *p* = 0.05) than super-killers. Thus, super-killers with broad inhibitory capacities had more restricted niches and grew less efficiently than non-inhibitory *Streptomyces*. This suggests that there may be significant tradeoffs between cumulative inhibition capacity and both niche width and growth efficiency among *Streptomyces.* Niche width reductions in super-killers may result from evolutionary tradeoffs between genomic investments in primary versus secondary metabolism, specifically reductions in the diversity of metabolic capacities retained by highly inhibitory populations. In contrast, reduced growth efficiencies among super-killers may be an ecological consequence of energetic costs associated with antibiotic production that limit population growth rates, rather than the diversity of metabolic pathways.

**Fig. 5:**
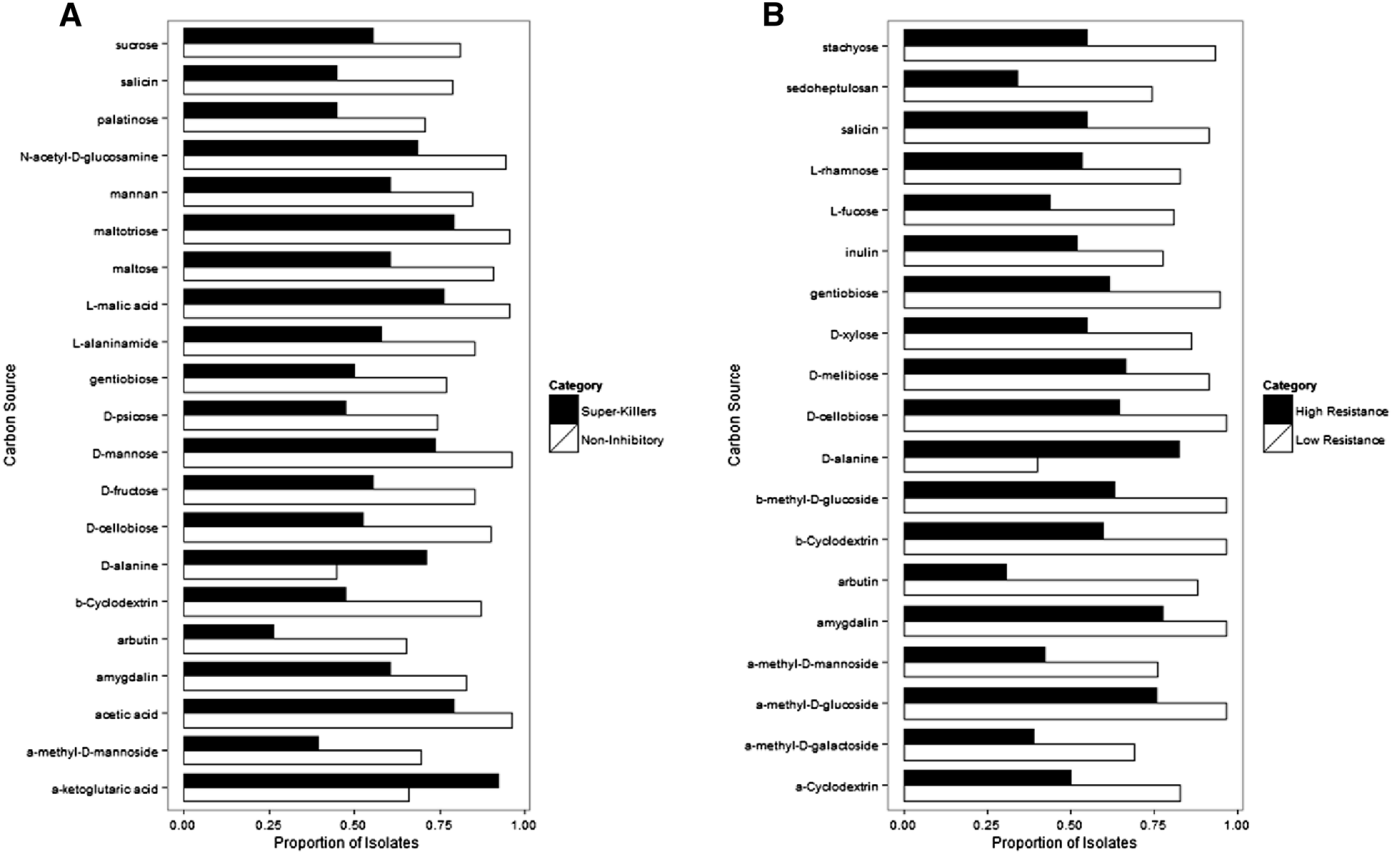
Sole carbon sources present on the Biolog SF-P2 plate that were used significantly more or less frequently among non-inhibitory versus super-killer *Streptomyces* (**a**) or those with low versus high resistance capacity (**b**). Significant differences in frequencies of carbon source use were determined with Pearson’s Chi-squared tests and, p-values <0.05 after FDR correction were considered significant

*Streptomyces* with distinct inhibitory capacities (super-killers versus non-inhibitors) differed in their likelihood of using specific carbon sources for growth. Of the 95 sole carbon sources present on the Biolog SF-P2 plate, 21 carbon compounds were used significantly more or less frequently by super-killers than non-inhibitory *Streptomyces* (Fig. 5a). These differentially used carbon sources included carbohydrates (*n* = 13), carboxylic acids (*n* = 3), amino acids (*n* = 2), polymers (*n* = 1), and miscellaneous compounds (*n* = 2). In general, super-killers used carbon sources less frequently than non-inhibitory *Streptomyces* (*n* = 19 of 21 differentially used sole carbon sources), consistent with a niche width-inhibition capacity tradeoff. However, D-alanine and α-ketoglutaric acid were used at significantly higher frequencies among super-killers than among non-inhibitory strains (71 % vs. 45 % and 92 % vs. 66 %, respectively).

### Resistance-resource use tradeoffs

*Streptomyces* that could resist inhibition by particular standards were sometimes less growth efficient or had smaller niche widths than isolates that were susceptible to that *Streptomyces* (Table 2). Isolates able to resist standards LK2-12, LK4-16, LK4-24, LK6-14, DL87, and DL93 were significantly less growth efficient than isolates that were susceptible to these standards. Similarly, *Streptomyces* that were able to resist standards LK4-16 and DL93 also had smaller niche widths than susceptible *Streptomyces.* In general, *Streptomyces* resistant to individual standards were consistently less growth efficient (9 of 10 standards) or had smaller niche widths (7 of 10 standards) than *Streptomyces* susceptible to that standard.

**Table 2:** Differences in growth efficiency and niche widths among *Streptomyces* susceptible and resistant to individual standards

Standard	Growth Efficiency (± SD)					Niche Width (± SD)				
	Resistant	Susceptible	df	t-val	p-val	Resistant	Susceptible	df	t-val	p-val
LK2-12	0.062 ± 0.013	0.068 ± 0.016	94.3	−2.40	0.018	72.6 ± 13.0	73.3 ± 17.2	104.1	−0.33	0.742
LK4-2	0.066 ± 0.015	0.068 ± 0.014	20.1	−0.39	0.701	72.8 ± 16.6	74.1 ± 15.5	20.1	−0.34	0.740
LK4-16	0.064 ± 0.014	0.079 ± 0.017	56.8	−5.85	<0.001	71.1 ± 16.8	81.9 ± 10.7	96.1	−5.47	<0.001
LK4-20	0.067 ± 0.015	0.059 ± 0.010	4.4	1.68	0.161	73.2 ± 16.5	72.0 ± 12.8	4.3	0.21	0.845
LK4-21	0.063 ± 0.013	0.067 ± 0.016	62.1	−1.66	0.103	73.3 ± 11.9	73.2 ± 17.2	75.3	0.04	0.965
LK4-24	0.063 ± 0.013	0.068 ± 0.016	130.5	−2.57	0.011	72.7 ± 12.7	73.3 ± 17.5	148.2	−0.32	0.748
LK6-14	0.066 ± 0.015	0.078 ± 0.006	3.6	−3.44	0.031	73.1 ± 16.5	76.8 ± 13.2	3.1	−0.54	0.625
LK10-3	0.066 ± 0.015	0.073 ± 0.009	3.3	−1.49	0.226	73.3 ± 16.5	75.5 ± 6.6	3.6	−0.65	0.555
DL87	0.066 ± 0.015	0.082 ± 0.003	1.5	−6.86	0.043	73.2 ± 16.4	67.5 ± 13.4	1.0	0.60	0.654
DL93	0.063 ± 0.013	0.081 ± 0.017	66.5	−7.03	<0.001	71.6 ± 16.3	80.9 ± 12.4	100.2	−4.51	<0.001

Growth efficiency (±standard deviation) and niche width (±standard deviation) of *Streptomyces* resistant and susceptible to inhibition by individual standard isolates. Degrees of freedom, t-values, and p-values of t-tests between resistant and susceptible *Streptomyces* are presented

In contrast to relationships for inhibition intensity, resistance zones (inhibition zone size of standards *Streptomyces* against environmental isolates) had no clear relationship with growth efficiency or niche width. This suggests that the costs of resistance may be largely associated with the presence of a resistant phenotype, rather than the degree of resistance conferred. However, the number of susceptible *Streptomyces* varied considerably for individual standards (*n* = 2 to *n* = 213; Additional file 1), which may limit our ability to detect significant relationships in cases where there are very few susceptible isolates.

The number of standards that *Streptomyces* isolates could resist was negatively correlated with niche width (Fig. 3; Spearman’s *ρ* = −0.26, *p* < 0.0001, *z* = −4.15) and growth efficiency (Fig. 3; Spearman’s *ρ* = −0.35, *p* < 0.0001, *z* = −5.72). *Streptomyces* with greater resistance capacity tended to have smaller niche widths and grow less efficiently than more susceptible isolates. *Streptomyces* with very high versus low antibiotic resistance capacities also differed significantly in resource use. Isolates with little resistance to inhibition by standards (those that could resist inhibition by ≤5 standards) had significantly larger niche widths and higher growth efficiency than highly resistant *Streptomyces* (those that resisted inhibition by ≥8 standards; Fig. 4). On average, *Streptomyces* with little resistance had 12.4 % larger niche widths (*t*-test, *t* = 4.12, *p* < 0.0001), and 28.0 % greater growth efficiency (*t*-test, *t* = 6.53, *p* < 0.0001) than *Streptomyces* with a high number of resistances, suggesting substantial growth efficiency-resistance and niche width-resistance tradeoffs among *Streptomyces* isolates. Tradeoffs between niche width and resistance capacity may reflect distinct genomic investment in primary metabolism versus antibiotic resistance strategies, where highly resistant isolates are less capable of metabolizing diverse carbon sources. In contrast, growth efficiency-resistance tradeoffs may be a consequence of the direct physiological costs of bearing multiple resistance traits that limit population growth rates.

*Streptomyces* with low versus high capacities to resist inhibition by standards differed significantly in the frequency with which they used specific carbon compounds for growth. Of the 95 sole carbon sources present on Biolog SF-P2 plates, nineteen carbon sources were used at different frequencies between *Streptomyces* with low versus high resistance capacities (Fig. 5b). Differentially used carbon compounds included carbohydrates (*n* = 13), polymers (*n* = 3), amino acids (*n* = 1), and miscellaneous compounds (*n* = 2). Similar to patterns observed for super-killers versus non-inhibitors, *Streptomyces* with low resistance capacities utilized most carbon compounds at significantly higher frequencies than those with high resistance capacities (*n* = 18 of the 19 differentially used sole carbon sources). Intriguingly, D-alanine was an exception and was used for growth by a greater proportion of highly resistant strains (82 % of strains) than those with low resistance capacities (40 % of strains). Together, these data indicate that reductions in niche width among *Streptomyces* with high versus low resistance capacities are often specific to individual carbon compounds.

## Discussion

Most *Streptomyces* were characterized by one of two distinct inhibitory strategies, tending to be either non-inhibitory, or highly inhibitory “super-killers”. Because antibiotic inhibitory interactions can be highly specific among *Streptomyces* isolates [4, 29], the benefits of producing a particular antibiotic may vary across communities. Specifically, since genes encoding resistance to antibiotics are broadly distributed in natural soil habitats [7, 16, 30], competitors may acquire resistance to a specific antibiotic and offset the benefits of antibiotic production to the producing organism [6, 13]. However, combinations of antibiotics, especially those with different modes of action, can substantially reduce the likelihood that any one competitor bears resistance to every antibiotic and may slow the evolution of novel resistances [31]. Indeed, selection for *Streptomyces* to produce antibiotic compounds that act synergistically is thought to play a significant role in the evolution of *Streptomyces* secondary metabolism [15]. Thus, accumulating multiple synergistic or complementary pathways for antibiotic production within individual *Streptomyces* strains may confer substantial and potentially synergistic fitness benefits to producers across time and space. As a result, optimal strategies for an antagonistic lifestyle may tend towards relatively high accumulation of antibiotic inhibitory phenotypes (super-killers), as observed here.

Although antibiotic phenotypes are assumed to impose costs of production [11–13] our explicit goal was not to determine physiological costs of *in vitro* antibiotic production but rather to document life-history tradeoffs across a diverse collection of naturally-occurring *Streptomyces* isolates. Negative relationships between *Streptomyces* inhibitory phenotypes and niche width or growth efficiency and the substantially smaller growth efficiencies and niche widths for super-killer versus non-inhibitory *Streptomyces* suggest significant growth-inhibition and niche width-inhibition tradeoffs associated with these traits. These tradeoffs may represent physiological costs of constitutive antibiotic production, pleiotropic effects of genes involved in primary or secondary metabolism, or the costs of accumulating antibiotic biosynthetic pathways for multiple antibiotic compounds.

Negative relationships between inhibition intensity against standards and growth efficiency or niche width were common, yet significant differences in growth efficiency or niche width between inhibitors and non-inhibitors were observed for only a few standards. This suggests that the physiological costs of inhibition of a single target may be more strongly associated with inhibition intensity, while genomic costs were more evidenced in cumulative inhibitory capacities. Physiological tradeoffs associated with expressing a given biosynthetic pathway for antibiotic production may be offset by tightly-controlled regulation. This is frequently the case among *Streptomyces*, where genes encoding the production of many secondary metabolites have diverse and highly complex regulatory mechanisms which often require very specific growth conditions, nutrient limitation, and/or extra-cellular signals for expression [32–35]. In general, the biosynthesis of many antibiotic compounds produced by *Streptomyces* occurs when nitrogen, phosphorus, or carbon sources become limiting and the process of morphological differentiation is triggered [23, 32]. Moreover, some specific carbon compounds, such as N-acetyl-glucosamine, can have differential effects on this process depending on resource availability [22]. Because of the complex and tightly-controlled nature of antibiotic biosynthesis, the tradeoffs observed in this work between inhibition intensity and growth efficiency or niche width may be capturing only a small subset of the potential antibiotic compounds produced by *Streptomyces* isolates. We observed growth efficiency and niche width tradeoffs associated with individual resistance phenotypes only for a small number of standards, suggesting that the cost of any specific resistance phenotype may be very small. This may be due in part to compensatory mutations, which are likely to minimize tradeoffs by rapidly alleviating the costs of antibiotic resistance [36, 37] or a low cost of resistance mechanisms that require induction by antibiotic compounds [38]. However, the significant negative correlation between accumulated resistance capacities with growth efficiency and niche width, as well as the substantial difference in growth efficiency and niche width among highly resistant versus susceptible isolates, suggests that costs of resistance can be substantial as multiple resistances accumulate. Although some resistance mechanisms may provide resistance to a broad range of antibiotic compounds with little or no additive cost (eg. multi-drug efflux pumps), epistatic interactions between distinct resistance mechanisms or resistance mutations are also suggested to be crucial to the fitness costs of antibiotic resistance [39, 40]. Thus, the greater-than-additive costs of multiple resistances are likely to be important constraints to the accumulation of antibiotic resistances within *Streptomyces* populations in soil.

In contrast to antibiotic inhibition, cumulative capacities to resist antibiotic inhibition (the number of standards that an isolate could resist) were approximately normally distributed among *Streptomyces*, and resistance to inhibition by an intermediate number of standards was the most common phenotype among isolates. In natural settings *Streptomyces* are commonly resistant to many antibiotics, even in environments with no history of antibiotic contamination from anthropogenic sources [16, 30]. This suggests that species interactions in natural populations contribute to the maintenance of multiple resistance phenotypes. In particular, strong selection for resistance is expected to result from the severe fitness consequences of being susceptible to inhibition by antibiotics produced by competitors. However, reductions in growth efficiency and niche width as antibiotic resistances accumulate may constrain the acquisition of greater resistance capacities in *Streptomyces* isolates. These constraints combined with the substantial fitness cost of lacking resistance to antibiotic-producers may generate stabilizing selection that favors *Streptomyces* with moderate resistance capacities rather than those having very few or very many antibiotic resistances.

Highly inhibitory super-killer and highly resistant *Streptomyces* used many carbon sources at significantly lower frequencies than non-inhibitory or poorly resistant *Streptomyces*. This suggests that reductions in niche widths associated with these lifestyles are resource-specific (Fig. 5), and that physiological or genomic costs of these life-styles are reflected in more restricted primary metabolic capabilities to utilize specific compounds for growth. In some cases (eg. for D-alanine, an essential component of the peptidoglycan layer of the bacterial cell wall), these compounds may be have a specific physiological role in antibiotic resistance phenotypes [41].

Although we found compelling evidence for resource use tradeoffs with antibiotic inhibition and cumulative resistance capacity, there was substantial variability in niche width and growth efficiency among *Streptomyces* with different antibiotic inhibition and resistance phenotypes. This may in part be due to the assumption that *Streptomyces* isolates able to inhibit or resist many other strains produce more antibiotic compounds or have a greater number of antibiotic resistance factors. However, the potential that for some *Streptomyces* a single antibiotic can inhibit multiple standards is likely to confound the relationship between cumulative inhibition capacity and niche width or growth efficiency among isolates. Furthermore, due to the complex regulation of antibiotic production, the phenotypic assays in this work likely captured only a subset of the inhibitory potential of our isolates. Similarly, the effectiveness of distinct resistance mechanisms for providing protection against multiple standards in this work is not known. Diverse mechanisms of antibiotic resistance (eg. efflux pumps, mutation, influx decrease, antibiotic modification) may confer resistance to multiple antibiotics, or may also impose different fitness costs [37, 40]. In light of these considerations, the substantial variation in niche width and growth efficiency among *Streptomyces* with similar inhibition or resistance capacities is not surprising. Further studies using population genomics tools to quantify the specific secondary metabolic pathways, resistance factors, and primary metabolic capacities of these populations will address these issues and offer critical insight into genomic and physiological tradeoffs among *Streptomyces*.

Intriguingly, reductions in growth efficiency and niche width were substantially smaller for *Streptomyces* with super-killer versus non-inhibitory life-history strategies than for those with highly resistant versus susceptible lifestyles. This suggests that it may be more costly to accumulate capacities to resist many antibiotics than to accumulate ability to inhibit (kill) many targets. This is a particularly novel finding considering that the fitness costs of antibiotic resistance in clinical settings are often found to be small [42] and that the pathways responsible for antibiotic biosynthesis require one or more large, multi-domain enzymes (polyketide synthases and non-ribosomal peptide synthases). Differences in costs of accumulating antibiotic resistance and antibiotic production pathways may contribute significantly to the dynamics of antibiotic inhibition and resistance in natural communities across the landscape.

## Conclusions

Our findings suggest that tradeoffs are likely to constrain the evolution of extreme antibiotic inhibitory and resistance phenotypes among natural populations of soil-borne *Streptomyces*. Moreover, greater apparent costs of accumulating multiple resistances versus inhibitory capacities are surprising, and appear to generate different distributions of these phenotypes among *Streptomyces* isolates. Further work exploring the roles tradeoffs play in the evolution of *Streptomyces* will be essential to elucidating the forces that generate and maintain the vast diversity of antibiotic inhibitory and resistance phenotypes in soil communities.

## Additional file

Additional file 1:
**Linear regression analysis of inhibition or resistance zone sizes with growth efficiency or niche width for only those** ***Streptomyces*** **able to inhibit or resist a given standard test isolate.** (PDF 270 kb)
